# The validity and psychometric properties of the Japanese version of the Compulsive Internet Use Scale (CIUS)

**DOI:** 10.1186/s12888-017-1364-5

**Published:** 2017-05-30

**Authors:** Roseline Kim Fong Yong, Akiomi Inoue, Norito Kawakami

**Affiliations:** 1 Department of Public Health, Graduate School of Medicine, Akita University, Akita, Japan; 2 Department of Public Health, Kitasato University School of Medicine, Sagamihara, Japan; 3 Department of Mental Health, Graduate School of Medicine, The University of Tokyo, Tokyo, Japan

**Keywords:** Internet addiction, Compulsive internet addiction behavior, Problematic internet behavior, Reliability and validity, Psychometric properties, Compulsive internet use scale, Internet gaming disorder (IGD), Behavioral addictions

## Abstract

**Background:**

Prolonged Internet use is often associated with reduced social involvement and comorbid psychopathologies, including depression, anxiety, attention-deficit/hyperactivity disorder, and obsessive–compulsive disorder. Asian countries where Internet access is widely available have high reported levels of Internet addiction. As Internet use has changed drastically since concerns about Internet addiction were first raised, the results of recent studies may be inaccurate because the scales they employed to measure Internet addiction were formulated for different Internet usage from the present. It is thus necessary to develop more-up-to-date scales to assess problematic private use of the Internet.

**Methods:**

The Compulsive Internet Use Scale (CIUS) was translated into Japanese. An online sample whose ages and sexes reflected that of the national population of Internet users was recruited to test the scale’s reliability and validity. Correlations between the scale and Internet-related parameters (such as time spent online, motivation for going online, and applications used) and psychosocial factors (such as psychological distress symptoms and loneliness) were examined. Psychometric properties were examined by the split-half method using both exploratory and confirmatory factor analysis. Model fits were compared across gender.

**Results:**

CIUS was found to have a high reliability and good concurrent, correlation and construct validity. Both exploratory and confirmatory factors revealed that the one-factor solution yielded a satisfactory result across gender. However, the three-factor structural model in which compulsiveness was gauged by “excessive absorption”, “difficulty in setting priorities”, and “mood regulation” gave the best fit of the model for the general population as well as across gender.

**Conclusions:**

Compulsive Internet behavior in Japan can be assessed in terms of absorption, priorities, and mood. CIUS is a valid scale for screening compulsive Internet behavior in the general Japanese population regardless of age and gender.

**Electronic supplementary material:**

The online version of this article (doi:10.1186/s12888-017-1364-5) contains supplementary material, which is available to authorized users.

## Background

Prolonged Internet use is often associated with reduced social involvement [1] and comorbid psychopathologies, including depression, anxiety, attention deficit hyperactivity disorder, and obsessive–compulsive disorder [2]. The fifth revision of the Diagnostic and Statistical Manual of Mental Disorders (DSM-V) classified excessive Internet gaming as Internet gaming disorder, including it in Section III of the appendix as a research diagnosis. Internet gaming disorder is characterized by preoccupation with Internet games, withdrawal, loss of control, continued use despite negative repercussions, lying to others, putting other things at risk for Internet games, as well as using Internet games as a way to escape guilt and anxiety [3]. Besides gaming, a number of other online activities, including participating in social network systems (SNS) and sexual and gambling sites, are thought to be addictive [4–9]. Overall, Internet addiction is found to be more prevalent among younger populations [7, 10, 11].

In general, men have been reported to be more likely than women to engage in problematic Internet use that involves seeking information, playing games, seeking fame, flirting, and participating in cyber-sex and cyber-pornography. Women are more likely to use the Internet to keep in touch with family, to seek support, friendship, and romance, and to complain about their partners [7, 12, 13]. However, some studies have found no gender differences for particular domains of addicted Internet use, such as free-to-play games [14].

The phenomenon has become a concern in Asian countries that have increasing Internet coverage, and the prevalence of Internet addiction has been reported to be higher in Asian countries than Oceania and European countries [15]. Surveys conducted in middle and high schools in Korea between 2006 and 2008 indicated that 1.6–10.7% of adolescents were severely addicted, while 29–38% were mildly addicted [16–18]. An earlier survey in Korea in 2003 indicated that 3.5% of the Korean Internet population experienced severe addiction and 18.4% show increased risk [19]. A previous study in 2000 reported that 8.1% of undergraduate students in Taiwan were Internet addicts [20]. In Hong Kong, a random telephone survey in 2004 indicated that 37.9% of adolescents were addicted to the Internet [21], while a random household survey in 2010 found that 6.7% of adolescents were addicted to the Internet [22]. Studies conducted between 2007 and 2012 in China among the middle and high school students indicated that 2.4–11% of adolescents were Internet addicts [23–25]. In Japan, where 78.2% of the population access the Internet according to a 2010 report [26], 2% of the population are estimated to be at risk of Internet addiction [27]. A 2011 study, which sampled city office employees between 22 and 55 years old, found that 6.1% of men and 1.8% of women in the studied population were pathological Internet users [28].

The reported prevalences are very high, which has prompted questions about whether Internet addiction is being excessively pathologized [29]. Researchers are beginning to question whether Internet addiction is an online manifestation of existing social problems. For example, online gambling, online pornography, and online bullying may just be a shift from an offline social problem to an online one, which may have nothing to do with Internet addiction [4, 30–32]. Attitudes to the Internet have also changed — a permanent Internet connection is generally considered essential for work. If a person is heavily dependent on the Internet because of official duties, their condition is really an online version of workaholism rather than Internet addiction. To classify Internet addiction correctly, we believe it is necessary to study the behavior by distinguishing between private Internet use and Internet usage for official duties. To better understand problematic Internet behavior in today’s environment where the Internet is ubiquitious, we believe that an up-to-date test model is required to screen for Internet addiction among the general population.

In the present study, we evaluated various scales for Internet addiction in terms of year of development, purpose, total items, and meaning of scores. Based on this assessment, we selected the Compulsive Internet Use Scale (CIUS) [33], which was developed at the beginning of the 2000s, when the Internet had become an important tool for both private and work activities. Meerkerk et al. [33] argued that assessments of Internet addiction should exclude Internet use for official duties. They examined the six criteria that Griffiths et al. [31, 34] formulated and the DSM-IV criteria for dependence and pathological gambling. They then conducted an explorative qualitative study on 17 self-declared Internet addicts contacted through an advertisement in a national newspaper. The interviews revealed that the most characteristic feature of Internet addiction were “inability to limit time spent online” and “preoccupation”, which expresses the tendency of the subjects to look forward to their next online session and to prefer using the Internet over social or leisure activities they had previously enjoyed. CIUS was developed based on these two main features [33]. The CIUS uses 14 items to assess compulsive Internet use, making it quick and convenient to combine with other scales. It seeks to measure compulsive Internet behavior of private Internet use only. Its 14 items assess the frequency of events (“how often…?”), with a total score ranging from 0 to 56 measured on a five-point Likert scale (“never”, “seldom”, “sometimes”, “often”, and “very often”) with good reliability (α = 0.89) and a good validity of its concise presentation of a one-factorial structure, “compulsiveness”, with an root-mean-square error of approximation (RMSEA) of 0.053–0.084 and a CFI of 0.966–0.984 [33]. CIUS has been used in a series of national population studies in the Netherlands [8, 33, 35], which demonstrated its suitability as a measurement scale. The scale has been translated into German, French, Icelandic, Arabic, and Chinese [36–40]. Validation of the French version on college students supported the one-factorial structure with a high internal consistency (Cronbach’s α = 0.91; RMSEA = 0.08; CFI = 0.92) [39].

In the present study, we translated the scale into Japanese and tested its psychometric properties on a Japanese group. Our aim was to evaluate the suitability of CIUS as a scale to measure compulsive Internet addiction in the general population across age and gender. CIUS was evaluated based on its normality distributions, reliability, criterion-related validity, and factorial structure. The validity of the structure was tested across gender.

## Methods

### Translation of CIUS

We obtained permission to translate CIUS into Japanese and use it in this study from the original author. We also obtained ethical approval from the Research Ethics Committee of Graduate School of Medicine of the University of Tokyo. All participants gave informed consent before taking part in the study. CIUS was translated using the forward and backward translation procedure [41]. Three bilingual native Japanese speakers (a Japanese professional translator, a Japanese occupational physician, and a Japanese licensed psychiatry social worker) did the forward translation and two bilingual native English speakers who were living in Japan (an American–Japanese sociologist and a Caucasian American information technology professional) did the backward translation. Two other bilingual native Japanese speakers (a Japanese research assistant who has a degree in translation and a Japanese psychiatric nurse) reworded the difficult questions in the reconciliation process. None of these translators were familiar with the scale. The face validity of the instrument was further confirmed by a team of Japanese psychiatric nurses, mental health social workers, and nursing students. The newly translated instrument was tested with different groups: teenagers, young adults, and adults. To increase overall acceptability, samples were recruited from the campus and personal networks. A total number of 42 samples were tested (ranging in age from 17 to 60 years, comprising students, jobless, and fulltime workers). Among them, eight samples were utilized for test-retest to clarify concept and examine consistency.

### Sample recruitment

To ensure a valid sample, we mimicked the national population of Internet users [42] in terms of sex and age (age 16–19 = 7.1% (men = 1154, women = 1158); age 20–29 = 16.3% (men = 2655, women = 2592); age 30–39 = 20.6% (men = 3487, women = 3426); age 40–49 = 19.8% (men = 3362, women = 3346); age 50–59 = 16.4% (men = 3068, women = 3127); age > 60 = 19.8% (men = 6934, women = 8765)). Samples were recruited from an Internet survey company, Macromill, which had a database of 1,086,904 registered users in May 2012. To ensure a sample size of 10 cases for each item to be factor analyzed, we asked the company to recruit 600 participants older than 16, pre-stratified by sex and age in the ranges 16–19 (men = 21, women = 21); 20–29 (men = 49, women = 47); 30–39 (men = 63, women = 63); 40–49 (men = 60, women = 60); 50–59 (men = 45, women = 51); and >60 (men = 67, women = 53). The registered users were stratified by sex and age. They were then assigned pseudorandomized numbers and sorted in order of this number, resulting in a randomized data list. An invitation email containing a link to the survey was sent to a total of 4886 registered users on the top of the randomized list of the respective sex-age stratified group, age 16–19 (men = 318, women = 264); age 20–29 (men = 882, women = 612); age 30–39 (men = 568, women = 568); age 40–49 (men = 396, women = 398); age 40–59 (men = 233, women = 234); age > 60 (men = 191, women = 222), with an estimated response rate of 8–10%. The respondents opened the survey by clicking the link in the email. Before starting the survey, participants were asked to read a cover letter and check a consent box to indicate their willingness to participate in the study. An effective sample was computed based on the number of participants that completed the survey. Entry to the online survey was on a ‘first come, first served’ basis; that is, the link to the survey was disabled once the quota for an effective sample size for the gender and age range had been reached. The total time to complete the survey was considered during the data-cleaning process. To make it convenient for working people and students to participate in the survey, invitation emails were sent at 9 am on Saturday 28 July 2012. The sampling quota was estimated to have been reached on the following Monday (approximately 48–52 h).

### Measures

Demographic data. Demographic data (sex, age, education level, occupational status, location of residence, residing alone or with others) were collected to compare with the national population of Internet users [26].

Internet addiction scales. The 20-items Japanese Internet Addiction Test (IAT), one of the earliest scales developed to test Internet addiction behavior for both private and public Internet use, was used to test CIUS’s concurrent validity. IAT employs a five-point Likert scale (1 = never; 2 = seldom; 3 = sometimes; 4 = often; 5 = very often) giving a total score in the range 20–100. Although the cut-off point for the general Japanese population had not been well validated, the ranges for Internet addiction were set to 20–39 (normal), 40–69 (mild), 70 and above (severe) respectively by referring to the standards set by the author of IAT [7]. In this survey, Cronbach’s Alpha amounted to 0.941.

The 14-items Japanese Compulsive Internet Use Scale (CIUS) employs a five-point Likert scale (0 = never; 1 = seldom; 2 = sometimes; 3 = often; 4 = very often) giving a total score in the range 0–56. CIUS does not have a predetermined cut-off score for Internet addiction. While a cut-off score of 28 was suggested since this score indicates that the specified Internet use behaviors occur on average at least ‘sometimes’, the author of CIUS suggested that the cut-off score should be carefully considered for respective countries. We divided the scores into three equal-width tiers (tier 1 = 0–18; tier 2 = 19–37; tier 3 = 38–56) to test the degree of Internet addiction by comparing the total scores with those for anxiety/depression and loneliness and to compare with the normality distribution of scores. Then, by using the receiver operating characteristic (ROC) curves, we compared the sensitivity versus specificity across a range of values for the ability to predict Internet addiction by referring to the three levels of Internet addiction suggested by IAT.

Internet-related features. Aspects such as Internet-access mode (via personal computer, tablet, smartphone, simple mobile phone, game device), reason for using the Internet (official use, stress release, killing time, communicating with others, meeting net-friends, seeking information, sharing interests, sharing or discussing personal problems, local SNS), Internet application used (romance, pornography, 2-Channel/BBS, blogging/SNS, information release/upload, digital download, P2P/FTP, online game, online survey, online banking, online shopping) [26]. Participants were also asked how many hours they spent online a week for private Internet use.

Psychological distress. A six-item psychological distress scale (K6) (α = 0.85) [43] was used to access symptoms of psychological distress. The frequency of each symptom was measured on a scale from 0 to 4 (0 = never; 1 = rarely; 2 = sometimes; 3 = often; 4 = always); a score of 5 points and above was taken to indicate the presence of psychological distress symptoms.

Loneliness. A 20-item UCLA loneliness scale (α = 0.87–0.91) [44] was used to access loneliness. It was measured on a four-point Likert scale (1 = never agree; 2 = somewhat disagree; 3 = somewhat agree; 4 = always agree) with a total score in the range 20–80.

### Statistical analysis

The results of participants who gave complete responses were subject to data analysis by SPSS 18 and AMOS 17. The frequency distribution of demographics was examined by comparing the participants with the national sample [26], and the normality of distributions of the scale scores was assessed to examine how well it fitted the tested population. The normality distribution was considered to be reasonably close to normal if the range of skewness and kurtosis fell between −1.0 and +1.0 [45]. The normality of the distributions of the scale scores was assessed for the whole sample, as well as for scores for categorized according to gender and age group.

Reliability of CIUS was reported using Cronbach’s alpha (α), together with the means, to determine the internal consistency for CIUS.

Pearson’s correlations among the reason for using the Internet, the application used, and compulsive Internet use scores were examined to find what kind of reasons and application used are correlated with higher compulsive Internet use scores. Construct validity was measured by comparing different levels of the Internet addiction scores with the mean scores of time spent online, K6 and the UCLA loneliness scale. Education level and occupational status were also examined for mean differences of compulsive Internet use scores. ROC curve analysis was performed to compare the sensitivity versus specificity across a range of CIUS scores for the ability to predict Internet addiction [46]. The scale was then subjected to factorial structure analysis.

Factorial structure was tested across gender. A split-half cross-validation method was employed, where the sample was randomly split into half exploratory factor analysis (EFA), was employed to develop the initial model on first half. It was then tested on the second half by using confirmatory factor analysis (CFA), consulting the root-mean-square error of approximation (RMSEA), the comparative fit index (CFI), and relative/normed chi-square (χ^2^/df) [47–49]. The average variance extracted (AVE) and construct reliability of each construct (latent variable) within the scale were also assessed for convergence validity [50].

## Results

### Participants’ demographics

A total of 4886 invitation emails were sent to prospective subjects (randomization was done by assigning a pseudo-randomized number to registered users stratified by age and sex). The sampling quota was reached 26 h after the email invitations were sent; a total of 623 effective respondents were obtained from the online sampling. When the demographic characteristics of the participants were compared with the national Internet user population (11), no significant differences were found in terms of age, sex, or location of residence. However, compared to the national population, our sample had a higher percentage of those residing alone (16.1% vs. 6.1%), higher Internet access by personal computer (97.4% vs. 84.7%) and lower Internet access by simple mobile phone (14.6% vs. 65.9%). 3% of the tested population reached the upper tier of CIUS (score 38–56) and 2% of the tested population reached the severe level of IAT (score 70–100). In general, time spent online did not show any significant moderate or strong correlation with reason for going online or application used. However, a significant moderate positive correlation was found between the number of Internet access modes and applications used, such as blogging (*r* = 0.370) and accessing digital content (e.g., iTunes and YouTube) (*r* = 0.338).

### Reliability

The 14-item CIUS had an excellent internal consistency of α = 0.931. The mean scores of items 1, 2, 3, 4, 7, 8, 9, and 12 were in the range 1.01–1.63, while the mean scores of item 5, 6, 10, 11, 13, and 14 fell in the range 0.62–0.91. The total correlation for all items was larger than 0.5, indicating that all items were measuring similar concepts and that deleting any item would not increase the reliability.

### Normality distribution of the scales

Compared to IAT, which is heavily skewed (1.4), CIUS was considered to be normally distributed (skewness = 0.725; kurtosis = 0.209), and the mean (14.8) was close to the median (14.0) indicating that the distribution was almost symmetric about the mean. The scale was found to best fit the age range 16–19 (skewness = 0.2). The normality distributions for the other age groups were recorded as 30–39 (skewness = 0.5), 20–29 (skewness = 0.6), 40–49 (skewness = 0.8), and 50–59 (skewness = 0.6, age > 60 (skewness = 0.9). The mean was close to the median, indicating that the distribution was almost symmetric about the mean, and the distribution patterns were the same with all age groups and sex. In contrast, IAT best fit the age range 16–19 (skewness = 0.6) and 20–29 (skewness = 0.9), while the other age ranges were heavily skewed (skewness >1). The normal distribution of CIUS fits better than that of IAT for both men (CIUS skewness = 0.6, IAT skewness = 1.2) and women (CIUS skewness = 0.8, IAT skewness = 1.7).

### Criterion-related validity

Table 1 shows the correlational relationship between the Internet used factors with the tested Internet addiction scales. A significant moderate correlation was found between CIUS score and reason for using Internet, including stress release (*r* = 0.476), killing time (*r* = 0.408), making friends (*r* = 0.324), sharing interest (*r* = 0.367), and discussing personal problems (*r* = 0.382). Among the various Internet applications, only 2-channel/BBS exhibited a significant moderate correlation with CIUS score (*r* = 0.393). Pearson correlations between CIUS score and K6 were significantly positive (*r* = 0.389), as were those between CIUS score and the UCLA loneliness scale (*r* = 0.277). Higher Internet scores for men had a higher correlation with accessing romance site (*r* = 0.266) than women (*r* = 0.042). Correlations between CIUS scores and online pornography, social network systems, and all other applications were similar for both men and women.

**Table 1:** Pearson correlations between the interested variables with the Japanese Compulsive Internet Use Scale (CIUS) and Internet Addiction Test (IAT)

	CIUS	IAT
Technology concern (TC)		
Total time spent on private Internet use	.279^a^	.282^a^
Number of ways for Internet access	.172^a^	.209^a^
TC1- motivation		
Official use	.095^b^	.125^a^
Stress release	.476^a^	.417^a^
Kill Time	.408^a^	.363^a^
Communication	.250^a^	.227^a^
Net-friends	.324^a^	.354^a^
Information Seeking	.135^a^	.141^a^
Sharing interest	.367^a^	.380^a^
Sharing/discussion of personal problems	.382^a^	.367^a^
Local SNS	.289^a^	.264^a^
TC2- Application		
Romance Site	.183^a^	.226^a^
Porno Site	.207^a^	.232^a^
2-Channel/BBS	.393^a^	.430^a^
Blogging/SNS	.272^a^	.320^a^
Information release/upload	.266^a^	.288^a^
Digital download	.266^a^	.292^a^
P2P/FTP	.206^a^	.189^a^
Online game	.187^a^	.200^a^
Online survey	0.052	0.044
Online banking	0.047	0.068
Online shopping	.205^a^	.189^a^
Mental Health		
K6 (Anxiety/Depression)	.389^a^	.450^a^
UCLA Loneliness	.261^a^	.284^a^

^a^Correlation is significant at the 0.01 level (2-tailed)

^b^Correlation is significant at the 0.05 level (2-tailed)

IAT scores were found to be different between men and women, age and prefectures, whereas no significance difference was found between the CIUS scores of men and women or between those for different prefectures of residence. Significantly higher mean scores for Internet addiction scales for both sexes were obtained for younger age groups (16–19 years old and 20–29 years old). Furthermore, scores increased in the order: people living alone; single people who have never had a romantic relationship, people who were dissatisfied with their current romantic relationship, despatched workers; students, people with either middle school or graduate school education level (Table 2). Although mean scores were high in these groups, the scores were below the upper tier of the CIUS scale.

**Table 2:** Comparison of the Japanese Compulsive Internet Use Scale (CIUS) and the Internet Addiction Test (JIAT) mean scores for participants’ demographic subgroups and Internet-related characteristics

		Total (%)	CIUS	IAT
			Mean (SD)	Mean (SD)
Sex	Men	323 (52)	14.8 (9.65)	34.5 (13.36)
	Women	300 (48)	14.8 (10.31)	32.2 (12.28)
			*p =* 0.925	*p* < .001
Age	16–19	44 (7)	21.3 (11.16)	42.6 (16.82)
	20–29	100 (16)	18.5 (11.13)	38.9 (16.12)
	30–39	130 (21)	15.7 (10.26)	34.1 (12.51)
	40–49	124 (20)	14.2 (9.32)	31.7 (11.35)
	50–59	100 (16)	12.2 (8.19)	30.2 (9.82)
	>60	125 (20)	11.4 (7.9)	29.2 (8.74)
			*p* < 0.01	*p* < .001
Areas	Kanto	243 (39%)	14.6 (10.18)	32.8 (12.45)
	Hokkaido	28 (4%)	14.3 (8.6)	32.5 (11.5)
	Tohoku	25 (4%)	16.8 (12.07)	36.8 (12.51)
	Chubu	90 (14%)	14 (9.98)	33.4 (13.73)
	Kinki	145 (23%)	15.5 (9.66)	34.1 (12.73)
	Chukoku	27 (4%)	20.1 (11.56)	39.4 (17.35)
	Shikoku	20 (3%)	11.7 (7.79)	28.9 (6.93)
	Kyushu	45 (7%)	12.9 (8.21)	31.6 (11.81)
			*p* = 0.300	*p* < .05
Education level	Middle school	8 (1)	20.9 (9.9)	33.5 (13.0)
	High school	189 (30)	14.1 (9.9)	32.6 (12.6)
	Vocational school	80 (13)	13.9 (9.4)	33.6 (13.1)
	Four-year university/college degree	309 (50)	14.8 (10.2)	33.2 (13.1)
	Graduate school	31 (5)	20.4 (7.7)	40.4 (11.9)
	Other	6 (1)	13.3 (8.3)	28.3 (4.3)
			*p* < 0.05	*p* = 0.056
Occupational status	Full-time worker	192 (31)	15.6 (9.9)	33.5 (12.8)
	Contracted worker	30 (5)	14.0 (10.0)	33.1 (11.1)
	Dispatched worker	13 (2)	18.0 (10.8)	37.9 (14.4)
	Part-timer	68 (11)	13.5 (9.5)	31.9 (12.1)
	Self-employed	25 (4)	10.9 (7.7)	31.1 (10.8)
	Freeter	32 (5)	10.6 (8.5)	32.0 (11.9)
	Housewife/husband	121 (19)	13.4 (9.2)	30.0 (10.7)
	Student	58 (9)	20.7 (10.7)	42.4 (16.0)
	Jobless and others	84 (13)	14.4 (10.2)	33.7 (13.1)
			*p* < 0.001	*p* < .001
Living	Living with others	523 (84)	14.4 (9.83)	23.7 (12.59)
	Living alone	100 (16)	17 (10.45)	36.9 (13.9)
			*p* < 0.05	*p* < .01

**Table 3:** Japanese Compulsive Internet Use Scale (CIUS) exploratory factor analysis on first random split half sample (*n* = 311)

		Factor		
Item	Item wording	1	2	3
Factor 1: Excessive Absorption				
CIUS-14	How often do you feel restless, frustrated, or irritated when you cannot use the Internet?	.819	−.028	−.004
CIUS-6	How often do you think about the Internet, even when not online?	.812	.022	.019
CIUS-10	How often do you prefer to use the Internet instead of spending time with others (e.g., partner, children, parents)?	.808	−.174	.051
CIUS-5	How often do others (e.g. partner, children, parents) say you should use the Internet less?	.617	.195	−.223
CIUS-11	How often do you rush through your (home) work in order to go on the Internet?	.516	.038	.164
CIUS-7	How often do you look forward to your next Internet session?	.478	.061	.213
Factor 2: Difficulties in setting priorities				
CIUS-13	How often do you think you should use the Internet less often?	.386	.209	.191
CIUS-12	How often do you neglect your daily obligations (work, school, or family life) because you prefer to go on the Internet?	.373	.230	.012
CIUS-2	How often do you continue to use the Internet despite your intention to stop?	−.135	.967	.040
CIUS-1	How often do you find it difficult to stop using the Internet when you are online?	.037	.875	−.067
CIUS-3	How often are you short of sleep because of Internet?	−.012	.681	.092
CIUS-4	How often have you unsuccessfully tried to spend less time on the Internet?	.236	.643	.000
Factor 3: Mood regulation				
CIUS-8	How often do you go on the Internet when you are feeling down?	−.048	.012	.973
CIUS-9	How often do you use the Internet to escape from your sorrows or get relief from negative feelings?	.054	.015	.876
	% Variance explained	46.4	7.8	6.5
	Cronbach’s α coefficient	.856	.877	.931
	Chi-square	122.323		
	df	52		
	*p*-value	.000		
	χ^2^/df	2.4		

In comparison to the three groups of proposed cut-off points for IAT, (20–39 = no addiction; 40–69 = mild addiction; 70–100 = severe addiction), CIUS was divided into three tiers of equal width (tier 1 = 0–18; tier 2 = 19–37; tier 3 = 38–56). Whereas IAT yielded a prevalence of 14 (2%) for its suggested highest score range, CIUS yielded a prevalence of 16 (3%) in its highest score range. Total time spent online was significantly lower for CIUS tier 1 (mean = 19.8; SD = 21.8), but did not differ much between tier 2 (mean = 32.9; SD = 26.1) and tier 3 (mean = 32.4; SD = 20.8). K6 scores were significantly lower for CIUS tier 1 (mean = 2.9; SD = 4.4) and increased with severity (tier 2: mean = 6.1, SD = 5.3; tier 3: mean = 7.8, SD = 7.7). UCLA loneliness scores were also significantly lower for CIUS tier 1 (mean = 40.8; SD = 11.9) and increased with severity (tier 2: mean = 45.8, SD = 11.6; tier 3: mean = 47.4, SD = 16.1).

ROC curve analysis showed that the highest proportion of congruent classified cases of the CIUS and the IAT (defined as the maximum sum score of sensitivity and specificity) was reached at a CIUS cut-off point of 18 and above for mild addiction (AUC = 0.938 (0.918–0.958), sensitivity = 0.905, specificity = 0.794), 23 and above for severe addiction (AUC = 0.989 (0.980–0.999), sensitivity = 1.0, specificity = 0.788).

### Exploring factor analysis

Measures of sampling adequacy for the first random split-half sample were carried out on the 14-item CIUS to see whether it was suitable for factor analysis. The correlation matrix showed a consistent pattern of inter-item correlations (0.304 < *r* < 0.701), with an exception of two relatively low inter-item correlations (item 3: 10, *r* = 0.269; item 5:8, *r* = 0.264) and two inter-item correlations that were considerably higher than others (item 1:2, *r* = 0.799, item 8:9, *r* = 0.872). Barlett’s test of sphericity yielded a chi-square value of 2696.9 (*p* < 0.001). A KMO of 0.915 indicated that factor analysis was appropriate. The basic Scree test did not show a clear break in the plots. However, when the criterion of eigenvalue >1.0 was used, three factors were generated from the CIUS. These three factors yielded a good fit to the data (χ^2^/df = 2.4) using maximum oblique rotation in five iterations. The three factors explained 60.7% of the variance. Item communality was found to be acceptable (0.315–0.901). Table 3 shows that factor 1 (6 items) accounted for 46.4% of the variance (α = 0.856) and appeared to measure the absorption of a person in the Internet activity (CIUS-Absorption). Factor 2 (6 items) accounted for 7.8% of the variance (α = 0.877) and appeared to measure the difficulties of the person in setting priorities (CIUS-priorities). Factor 3 (2 items) accounted for 6.5% of the variance (α = 0.931) and appeared to measure the use of Internet for mood regulation (CIUS-MR). All three factors appeared to correlate moderately with each another (*r* = 0.563–0.665).

### Confirmatory factor analysis

**Fig. 1:**
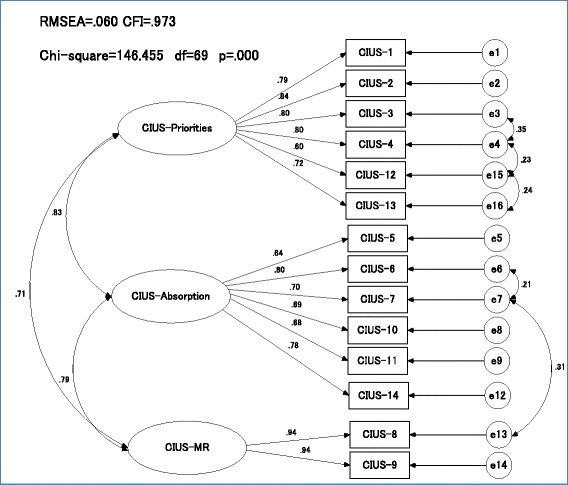
Japanese Compulsive Internet Use Scale (J-CIUS) confirmatory factor analysis on 2nd random split half sample (*n* = 311). Note: RMSEA: Root mean square error of approximation (<.05 excellent fit, 0.05–0.06 good fit, 0.07–0.08 adequate fit, 0.09–0.1 fair fit, >0.1 poor fit). CFI: Comparative Fit Index (≧ .95 good fit)

The factorial structure of CIUS was then examined using CFA utilizing maximum likelihood estimation with robust standard errors and a mean-adjusted chi-square statistic test on the second random split-half sample (*n* = 312). The three-factor solution showed adequate convergent validity (AVE = 0.51–0.88), a high construct reliability (α = 0.86–0.94), and a good fit (RMSEA = 0.06; CFI = 0.973; χ^2^/df = 2.1) by allowing the errors of a few items (items 3 and 4; items 4 and 12; items 6 and 7; item 7 and item 7) to co-vary, as indicated by the modification indices (Fig. 1). The covariance of these errors was justified as the items within construct were very closely related; the original developer suggested a similar error covariance. Correlations between factors were found to be relatively high (*r* = 0.713–0.831). The three-factor solution was tested with the full sample and for men and women separately. It showed an adequate fit (RMSEA = 0.074–0.086; CFI = 0.939–0.959; χ^2^/df = 2.7–4.4). A one-factor solution suggested by the original developer was also tested to compare its model fit with the three-factor solution. It gave an adequate fit (RMSEA = 0.079; CFI = 0.952; χ^2^/df = 3.0) when similar errors of the items were allowed to co-vary. The model gave a relatively good fit when tested with the full sample, the male sample, and the female sample (RMSEA = 0.085–0.094; CFI = 0.935–0.942; χ^2^/df = 3.7–5.5).

## Discussion

Our study showed that CIUS is compatible with IAT in terms to its concurrent validity and construct validity. However, as IAT is heavily skewed towards younger people, CIUS is a better scale than IAT for measuring problematic compulsive Internet behavior in the general Japanese population. The normal distribution results for IAT were only good for people below 30, and it is very skewed with either men or women in the general population (skewness >1.0). In contrast, the normal distributions for CIUS fall into acceptable ranges for all ages, even for elderly people (skewness <1.0). The significant mean score differences for gender and living locations for IAT are also a concern as they seem to be biased. In contrast, CIUS does not show these characteristics.

The current study sought to assess the psychometric properties of CIUS in the Japanese general population. As EFA and the eigenvalue suggested the three-factor solution, we compared the one-factor solution with the three-factor solution using CFA. Similar to previous studies [33, 36–40], the one-factor solution showed acceptable validity for both men and women, as well as in the general population. However, we found that the three-factor solution gave the best fit for the Japanese population across sex. The convergent validity implies that the three factors are distinct from each other. We conclude that the scale represents compulsiveness as a whole, where “compulsiveness” in Japan can be recognized as a combination of “excessive absorption”, “difficulties in setting priorities,” and “mood regulation”. In reference to the results of the construct validity where we had found a higher correlation between stress leases, killing time and CIUS scores, “compulsiveness” as a “mood regulation” seems to be self-explanatory. In line with previous studies [33, 36–38, 40, 51], correlated error pairs were found between items 6 and 7 and between items 12 and 13. In addition, we found correlated error pairs between items 4 and 12 and between items 7 and 8, which have not been reported elsewhere. These correlated error pairs suggest that these items overlap to some extent and that one member of the pair could be omitted in a shorter version of CIUS. Although the factorial structure found with both EFA and CFA were acceptable for the general population regardless of gender, our sample size is too small to test whether the same psychometric properties are applicable to different age groups.

In the current definition of IAT, the addictive level is presumably set to be above a score of 70, which yielded a prevalence of 14 (2%) of severe addicts. The results in our study shared similarities with the Canadian study on high school students using the same cut-off [52]. As CIUS does not have a preset cut-off point, we used three equal tiers; the highest tier (scores 38 and above) yielded a prevalence of 16 (3%) of severe addicts, resulting in a higher prevalence compared to IAT. Although the question to which level of the score where a person is considered severe addictive or problematic compulsive behavior with Internet cannot be answered by simply dividing the scores into three levels, the higher the scores of the equal tiers were, the higher the scores for psychological distress and loneliness were observed.

In this study, the ROC curve analysis allowed us to suggest cut-off points for CIUS by referring to the presumable addiction level determined by IAT, scores 18 and above as mild addiction and scores 23 and above as severe addiction. Both cut-off points had very high sensitivity (>0.9) and specificity (>0.8). The cut-off point for mild addiction identified in this study is similar to a previous German study that sought to determine a threshold for CIUS which matches the IAT cut-off for detecting problematic Internet use [53]. These cut-off points are useful in general population screening for Internet addiction, yet they should not be taken as valid diagnostic cut-offs because the scores were not oriented toward a specific behavior (e.g., gaming, pornography). In addition, the possibility of using IAT as a gold standard for Internet addiction is still being debated.

Higher psychological distress symptoms, feeling of loneliness, and excessive time spent on private Internet use were found in the middle and highest levels of Internet addiction scores, which concurs with previous findings [5, 7, 10, 30]. This confirms the correlation validity of CIUS and suggests that Internet addiction is a serious health threat and may indicate poor mental health.

One merit of this study is that it imitates the national Internet-user population by matching sex, age and region of residence. As we had a lower rate of those who accessed the Internet through mobile phones than the national Internet-user population, many in our sample do not solely rely mobile phones for Internet access.

Similar to previous studies, this study found that the number of hours spent on the Internet [9, 33] does not accurately reflect problematic Internet behavior unless they are due to relationship difficulties or poor performance at school or work. The Japanese translation of CIUS can gauge Internet behavior better than other scales, but to better define problematic behavior, future studies should consider assessing the probability of compulsive Internet behavior inducing poor relationships, unproductive time at work, loss of time with family and friends as well as spent on hobbies and interests.

Although the score distributions are close to normal across age, the samples were too small to examine the validity of factorial structure in each age group. Since it is widely thought that Internet addiction differs with age, future studies should use sufficiently large samples in the different age groups to permit a separate analysis to be conducted to check whether different generations have different Internet addiction patterns. It would also be beneficial for a longitudinal study to examine the test-retest reliability and the consistency of the construct and factorial validity of the scale.

### Strengths

The strength of this study is the pre-stratification of sex and age and pseudo-randomization during sample recruitment. Consequently, the sample is considered to be quite representative of the national population in terms of demographic distribution. Since the factorial structures of the three scales were performed on the whole sample as well as on men and women separately, the results of the study are applicable across sex. The assured anonymity of participants could have reduced reporting bias.

### Limitations

There were several methodological limitations. The sample was recruited through an Internet survey company, which limits participants to people registered with the company. Since recruitment was performed on a weekend and the survey could only be completed on a personal computer or laptop (although participants may have received invitations on their mobile phones), our sample may include many who prefer to spend their weekends online rather than going out or meeting friends and family. Also, people who reside alone are overrepresented, which may limit the generalizability of the results. A sampling bias may have been introduced by the fact that the survey could only be accessed through PC (although invitations were equally distributed to mobile and PC registered users). For that reason, it probably underestimates the number of Internet users that heavily depend on smartphones for accessing the Internet. Also, people living alone probably have more time to do an Internet survey on a Saturday than those living with others. As for the cut-off for both IAT and CIUS had not been well validated, the presentation of prevalence of Internet addiction in this study should be considered cautiously.

## Conclusions

The validity and psychometric properties of CIUS demonstrate its suitability as a screening tool for compulsive Internet behavior among all age groups in the Japanese population. Furthermore, it is valid for both men and women. The next step is to determine cut-off points for levels of addiction. A larger sample size is needed to compare factorial structures between young and older people. The effect of Internet addiction should be further explored by examining the relationship between high scores and the impact of Internet addiction on daily living.

## Additional file

Additional file 1: Translation and back translation of Japanese version of Compulsive Internet Use Scale (CIUS). (XLSX 13 kb)
